# Making sense of snapshot data: ergodic principle for clonal cell populations

**DOI:** 10.1098/rsif.2017.0467

**Published:** 2017-11-29

**Authors:** Philipp Thomas

**Affiliations:** Department of Mathematics, Imperial College London, London SW7 2AZ, UK

**Keywords:** stochastic gene expression, population dynamics, population snapshots

## Abstract

Population growth is often ignored when quantifying gene expression levels across clonal cell populations. We develop a framework for obtaining the molecule number distributions in an exponentially growing cell population taking into account its age structure. In the presence of generation time variability, the average acquired across a population snapshot does not obey the average of a dividing cell over time, apparently contradicting ergodicity between single cells and the population. Instead, we show that the variation observed across snapshots with known cell age is captured by cell histories, a single-cell measure obtained from tracking an arbitrary cell of the population back to the ancestor from which it originated. The correspondence between cells of known age in a population with their histories represents an ergodic principle that provides a new interpretation of population snapshot data. We illustrate the principle using analytical solutions of stochastic gene expression models in cell populations with arbitrary generation time distributions. We further elucidate that the principle breaks down for biochemical reactions that are under selection, such as the expression of genes conveying antibiotic resistance, which gives rise to an experimental criterion with which to probe selection on gene expression fluctuations.

## 1. Introduction

Exploring the consequences of cell-to-cell variability is crucial to understand the functioning of endogenous and synthetic circuitry in living cells [1–4]. In clonal cell populations, differences between cells arise from several dynamical effects. An important source of heterogeneity is the intrinsic stochasticity in biochemical reactions [5–7]. Equally important, but often overlooked, is the substantial variability of individual cell division timings. In fact, two sister cells do not divide at the same time [8–10], which leads to more heterogeneous cell ages in clonal populations. Although both of these factors have been studied independently, their contributions cannot easily be separated when cells are growing and dividing [11,12]. Approaches that allow to investigate the interplay of these effects remain widely unexplored.

Stochasticity in the levels of molecules per cell is commonly modelled using the stochastic simulation algorithm [5,13]. While this approach fares well for non-growing cells, it does not take into account the fact that molecule levels per cell need to double over the cell division cycle, at least on average. A number of studies therefore considered lineages that track the biochemical dynamics inside a single dividing cell over many generations [14–19]. Such approaches are well equipped to model the statistics observed in mother machines [20,21], for example, or using cell tracking [22] that allow monitoring intracellular biochemistry in isolated cells *over time*. Modelling approaches that also account for the substantial variations observed in cell division timing are only currently being developed [23,24]. Generation times of single *Escherichia coli* [10] and budding yeast cells [25], for example, vary up to 40% and 30% from their respective means, and similar values have been observed in mammalian cells [26].

On the other hand, population snapshots are commonly used to quantify heterogeneity *across* clonal cell populations. Such data are obtained from flow cytometry [27] or smFISH [28], for instance. An important source of heterogeneity in these datasets stems from the unknown cell-cycle positions [29]. Sorting cells by physiological features—such as using cell-cycle markers, DNA content or cell size as a proxy for cell-cycle stage—are used to reduce this uncertainty [27,30,31]. It has also been suggested that simultaneous measurements of cell age, i.e. the time interval since the last division, could allow monitoring the progression of cells through the cell cycle from fixed images [30–33]. Presently, however, there exists no theoretical framework that addresses both cell-cycle variability and biochemical fluctuations measured across a growing cell population, and thus we lack the principles that allow us to establish such a correspondence.

In applications, it is often assumed that the statistics observed over successive cell divisions of a single cell equals the average over a population with marked cell-cycle stages at a single point in time [34]. In statistical physics, such an assumption is referred to as an ergodic hypothesis, which once it is verified leads to an ergodic principle. Such principles certainly fare well for non-dividing cell populations, but it is less clear whether they also apply to growing populations, in particular, in the presence of fluctuating division times of single cells. While this relationship can be tested experimentally [35,36], we demonstrate that it is also amenable to theoretical investigation.

In this article, we develop a framework to analyse the distribution of stochastic biochemical reactions across a growing cell population. We first note that the molecule distribution across a population snapshot sorted by cell ages disagrees with the statistics of single cells observed in isolation, similarly to what has been described for the statistics of cell-cycle durations [8,37,38]. We go on to show that a cell history, a single cell measure obtained from tree data describing typical lineages in a population [39–43], agrees exactly with age-sorted snapshots of molecule numbers. The correspondence between histories and population snapshots thus reveals an ergodic principle relating the cell-cycle progression of single cells to the population. The principle gives important biological insights because it provides a new interpretation to population snapshot data.

In the results, we investigate the differences of the statistics of isolated cell lineages and population snapshots. Section 2.1 develops a novel approach to model the stochastic biochemical dynamics in a growing cell population. We derive the governing equations for an age-sorted population and formulate the ergodic principle. In §2.2, we demonstrate this principle using explicit analytical solutions for stochastic gene expression in forward lineages and populations of growing and dividing cells. Our results are compared with stochastic simulations directly sampling the histories of cells in the population. Finally, in §2.3, we elucidate using experimental fluorescence data of an antibiotic-resistance gene that testing the principle allows us to discriminate whether a biochemical process is under selection.

## 2. Results

**Figure 1.**
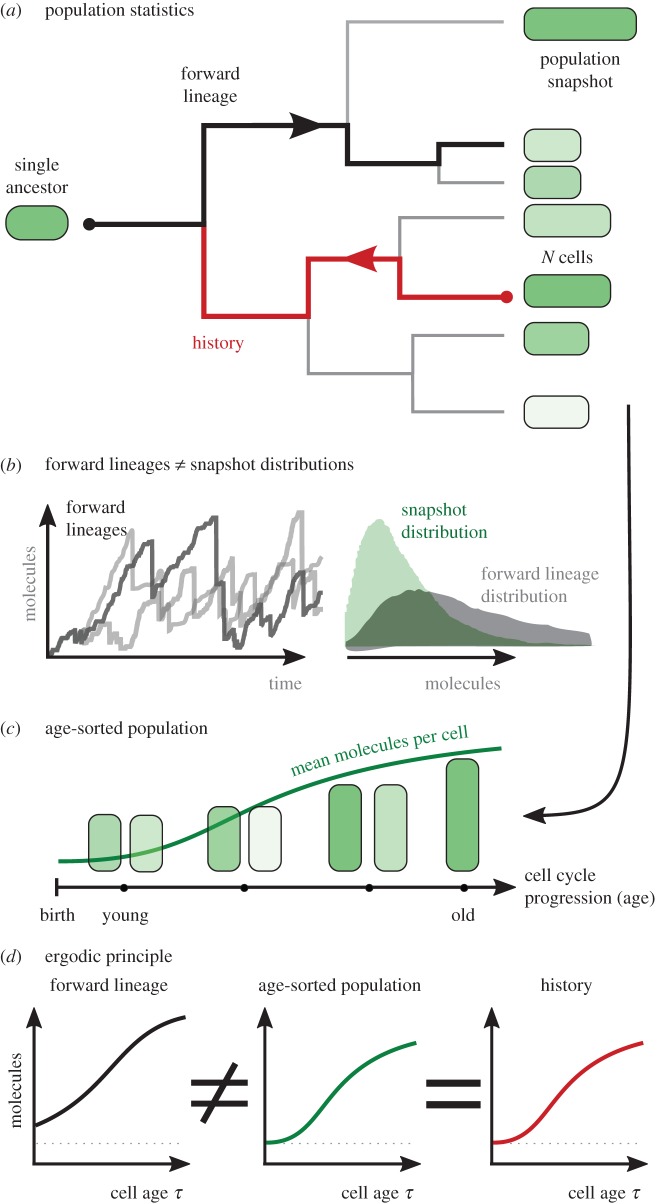
Ergodic principle between single cells and the population. (*a*) Lineage tree of a clonal population leading to heterogeneous distribution of molecule numbers per cell (green colour). Two types of lineage statistics characterize the tree: (i) *forward lineages* (black line) originate from a common ancestor, end at an arbitrary cell in the population, and (ii) *histories* start from an arbitrarily chosen cell in the population and end at a common ancestor (red line). The conceptual difference between these measures are the probabilities with which these lineages are selected. (*b*) Statistics of simulated forward lineages do not agree with snapshot distributions (see box 1 and caption of figure 5 for simulation details). (*c*) Statistics observed across the population and the statistics after sorting cells by age, which allows inferring the cell-cycle progression. (*d*) The ergodic principle states that the statistics of age-sorted cells equals the distribution observed along cell histories but not along forward lineages.

Several statistical measures can be used to quantify the levels of gene expression in single cells and populations. Distributions obtained across a cell population, such as those taken from static images, represent the final state of a growing population (figure 1*a*, shaded green cells). Cells in isolation, by contrast, can be modelled as random paths in the lineage tree, denoted as *forward lineages* (figure 1*a*, black line), that follow either of the two daughter cells with equal probability. Stochastic simulations show that the distributions of these two statistics disagree (figure 1*b*), and thus, apparently, the population dynamics violates the ergodic hypothesis. In the following analysis, we provide a novel ergodic principle relating how single cells progress through the cell cycle to the distribution of a growing cell population. To formulate the principle, we introduce *histories* (figure 1*a*, red line), a single-cell statistic that has the same distribution as a population that is sorted by cell ages (figure 1*c*,*d*). Such histories are obtained by choosing an arbitrary cell in the population and tracing its evolution back to the ancestor from which the whole population originated.

### 2.1. Biochemical dynamics in cell populations

We model stochastic biochemical reactions in a population of growing and dividing cells. We assume that each cell contains a pool of interacting biochemical species *X*_1_, *X*_2_, …, *X*_*N*_S__ reacting via a network of *R* intracellular reactions of the form
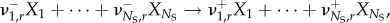
where *r* = 1, …, *R* and *ν*^±^_*j*,*r*_ are the stoichiometric coefficients. To model the effect of cell divisions, we associate to each cell an age *τ* measuring the time interval from cell birth. If cells divide with an age-dependent rate *γ*(*τ*), which is independent of the number of molecules *x* in the network under consideration, the division times *τ*_d_ of each cell are distributed according to2.1
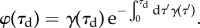
Equivalently, for a given division-time distribution one obtains the corresponding rate function via
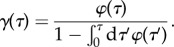


While the forward-lineage approach is summarized in appendix A, we here focus on the population dynamics. To this end, we associate with the state of the population the density of cells *n*(*τ*, *x*, *t*) with molecule count *x* and age between *τ* and *τ* + d*τ* at time *t*. This quantity represents the outcome of repeated snapshots of the population growth process, which averages out variations in the total number of cells (see appendix B for a detailed derivation). The mean number of cells in the population is then given by2.2
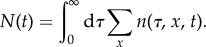
Since the probability for a cell to divide at age *τ* is given by *γ*(*τ*) d*τ*, the rate of change in the cell density obeys2.3*a*
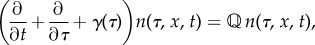
where 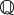 is the transition matrix for the biochemical reactions2.3*b*
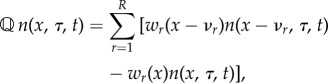
*w*_*r*_ is the propensity and (*ν*_*r*_)_*i*_ = *ν*^+^_*ir*_ − *ν*^−^_*ir*_ is the stochiometric vector of the *r*-th reaction. Equation (2.3*a*) must be supplemented by a boundary condition accounting for cell divisions. Because the number of newborn cells equals twice the number of dividing cells, the condition reads2.3*c*
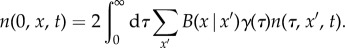
The division kernel *B*(*x* | *x*′) in equation (2.3*c*) models the inheritance of *x* daughter molecules from *x*′ mother molecules and is given by2.4
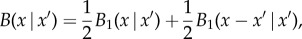
where *B*_1_(*x* | *x*′) and *B*_1_(*x*′ − *x* | *x*′) are the marginal distributions of inherited molecules for the two daughter cells (see appendix B for details). Note that the total molecule numbers are conserved during cell division. For example, when molecules are partitioned with equal probability between daughter cells, *B*_1_ and *B* are binomial [16,44].

A simple algorithm that enables simulating the biochemical dynamics in the population exactly, which we will refer to as the First Division Algorithm, is given in box 1. Step 2 simulates the transitions due to biochemical reactions, equation (2.3*b*), while step 3 implements the boundary condition (2.3*c*) for cell divisions. The density of cells with molecule count *x* and age *τ* obtained from several snapshots then obeys equations (2.3).

Box 1: **First Division Algorithm to simulate a cell population up to time *t*_*f*_.**

1. *Initialization.* At time *t* = 0, initialize the cell population by assigning to each cell an age *τ*_*i*_, a division time *τ*_d,*i*_ and molecule count *x*_*i*_.
2. *Biochemical reactions.* Determine the next dividing cell from *j* = argmin_*i*_ (*τ*_d,*i*_ − *τ*_*i*_) and Δ*t* = min_*i*_ (*τ*_d,*i*_ − *τ*_*i*_). Advance the molecule numbers of each cell independently from age *τ*_*i*_ to *τ*_*i*_ + Δ*t* using the Gillespie algorithm and advance time from *t* to *t* + Δ*t*.
3. *Cell division.* Replace the dividing cell by two newborn daughter cells of zero age. Assign to one of these a molecule number distributed according to *B*_1_(*x* | *x*_j_), depending on the mother's molecule count *x*_*j*_, and assign the remaining molecules to the other daughter. Assign to each daughter independently a division time distributed according to *φ*(*τ*_d_).
4. *Repeat.* Repeat from 2 until *t* = *t*_*f*_.

While stochastic simulations are relatively easy to carry out, equations (2.3) are generally difficult to solve because they represent an integro-partial differential equation. To allow for analytical progress, we consider the long-term behaviour in which the population grows exponentially with rate *λ*,2.5
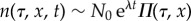
and identify *Π*(*τ*, *x*) as the joint distribution of cell ages and intracellular molecule counts in the population. We treat this distribution synonymously with the population snapshot. Using ansatz (2.5) in equation (2.3), we obtain2.6
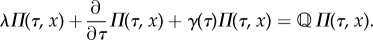
Similarly, the corresponding boundary condition is given by equation (2.3*c*) but with replacing *n*(*τ*, *x*) by *Π*(*τ*, *x*).

#### 2.1.1. Age distribution in a population

The age distribution measures the fraction of cells in the population that reach a given age. It is obtained from marginalizing the population distribution over the molecule numbers2.7
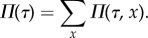
Summing equation (2.6) and solving (see appendix C for details), we find that the age distribution obeys2.8
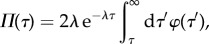
which recovers the results in [8,9,45]. The integral in the above equation denotes the probability that a cell has not divided before reaching age *τ*.

The population growth rate *λ* needs to be determined from the boundary condition (appendix C), which leads to the characteristic equation2.9
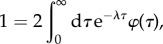
also known as the Euler–Lotka equation [8,9,45]. The largest root of this equation yields the population growth rate *λ*. For a discussion of the relation between the population growth rate and the mean division time, see [10,45].

#### 2.1.2. Molecule-number distribution in an age-sorted population

Next, we consider the probability of observing *x* molecules in a cell of age *τ*. This conditional probability is given by the number of cells with age *τ* and molecule count *x* divided by the number of cells at that age2.10
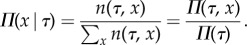
It can be verified by plugging equation (2.10) into (2.6) that the distribution obeys the master equation2.11*a*
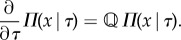
Similarly, inserting equation (2.10) with (2.5) into boundary condition (2.3*c*), we deduce that the distribution of inherited molecules obeys2.11*b*
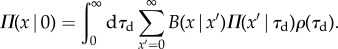
We identify the density *ρ* with the ancestral division-time distribution [8,40]2.11*c*
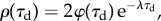
describing the past division-times in a growing cell population. Taken together, equations (2.11) describe the distribution in an age-sorted population. Combining these equations with the age distribution given in the previous section they contain the full statistical information of the population distribution. An extension of this result to heritable division times is provided in appendix C.

**Figure 2.**
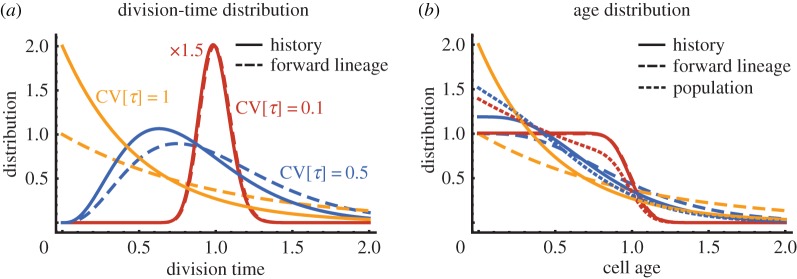
Generation time variability in forward lineages and histories. (*a*) Division times in forward lineages (dashed lines) following a gamma distribution with unit mean and coefficients of variation of 10% (red, scaled by factor 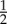), 50% (blue) and 100% (yellow dashed line). The corresponding division time distributions in cell histories are shown by solid lines. (*b*) The corresponding age distributions in forward lineages (dashed), histories (solid lines) and the population (light dashed). Note that the age-distribution in a history is the same as the population for the exponential distribution (CV 100%, yellow line), and the distributions in histories and forward lineages are very similar for small cell-cycle variability (CV 10%, red lines).

Since histories contain only ancestral cells, their division times are distributed according to the ancestral division-time distribution *ρ*, as has been pointed by Wakamoto *et al.* [40]. In figure 2*a*, we compare the division-time distributions in forward lineages and histories for variable division times. We observe that the distribution tails of *φ* are suppressed in cell histories due to exponential dependence on the growth rate *λ*. Specifically, we highlight the fact that cells with division times shorter than ln 2/*λ* are over-represented in histories compared with forward lineages, while cells with longer division times are over-represented.

#### 2.1.3. Ergodic principle for cell populations

We now explain how these quantities can be computed from population data. To this end, we use the approach presented by Nozoe *et al.* [42] enumerating *all* lineages of a given population tree. We denote the molecule numbers in lineage *j* by (*x*_1,*j*_(*τ*_1,*j*_), …, *x*_*D*,*j*_(*τ*_d, *j*_)) with *D*_*j*_ completed cell divisions, where *x*_*i*,*j*_(*τ*) is the time-course from birth to division of molecule numbers of the *i*th cell in that lineage. We then evaluate the average of a function *f*(*x*) over a lineage of cells at a given age *τ*,2.12
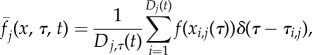
where 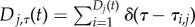 is the number of cells in the lineage that reach age *τ* before dividing.

Because forward lineages track either one of the two daughter cells with equal probability, the probability of choosing lineage *j* from the tree is 2^−*D*_*j*_^, which decreases with the number of cell divisions [42]. Because the division times in forward lineages follow the distribution *φ*, we have2.13
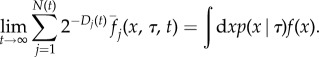
The distribution under the integral is the lineage-probability described in appendix A.

Next, we consider the distribution along histories that track an arbitrary cell from the population and trace back its evolution. The probability of choosing such a history is the same for every lineage and therefore equals the inverse number of cells *N*(*t*) in the population. The statistics of histories can thus be interpreted as a typical lineage. We note that equations (2.11) can be understood as describing the distribution along a lineage, similar to the forward-lineage approach develop in appendix A (cf. equations (A 1)), but by replacing the division-time distribution *φ* with the one of the ancestral population *ρ*. Since histories are composed entirely of ancestral cells, their division times follow the ancestral division-time distribution *ρ*. It hence follows that histories posses the same statistics as in the age-sorted population, that is2.14
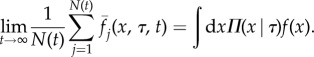
The distribution *Π*(*x* | *τ*) is the lineage distribution given by the solution of equations (2.11) and equals the distribution in an age-sorted population, as we have shown in §2.1.2. We thus formulate the ergodic principle as *the average of the histories of single cells in a population obtained over many cell divisions equals the average over an age-sorted cell population at every time point*. A more intuitive way of stating the result is that the endpoints of all lineages have the same distribution *Π*(*x* | *τ*) as cells of *τ* in the population. Note that equations (2.13) and (2.14) are equal only for deterministic division times. In fact, most lineages 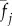 are close to the history-average given by the right-hand side of equation (2.14), as we show in the following section through the use of examples (§2.2).

Before proceeding, we investigate the age distribution that yields the fraction of cells reaching age *τ* in a history. Because division times in histories are distributed according to *ρ* and the probability that a cell has not divided before age *τ* is 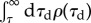, we have2.15
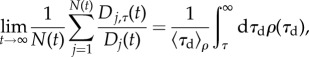
where 〈*τ*_d_〉_*ρ*_ is the mean of the distribution *ρ*, which arises as a normalizing constant (see appendix D). The result is notably different from the age-structure in a population *Π*(*τ*), compare equation (2.8). For example, the corresponding age distributions for gamma-distributed division times are compared in figure 2*b*.

It thus follows that the average over a history of molecule numbers is not generally the same as the population average irrespective of age. The only exception to the rule are memoryless division times, i.e. constant division rates resulting in exponentially distributed division timings. In this case, equation (2.8) and (2.15) agree, but interestingly, the population distribution differs from the forward-lineage distribution.

### 2.2. Analytical solutions for forward lineages, histories and snapshot distributions

To obtain analytical solutions we make use of the generating function approach. For this purpose, we restrict ourselves to binomial partitioning of molecules2.16
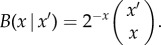
The boundary condition (2.11*b*) for the probability generating function 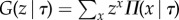 then simplifies to2.17
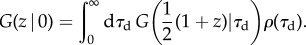
In the following, we demonstrate how explicit solutions for the lineage, histories and snapshot distributions can be obtained for simple examples of stochastic gene expression but arbitrary division-time distributions.

#### 2.2.1. Molecule synthesis and degradation

**Figure 3.**
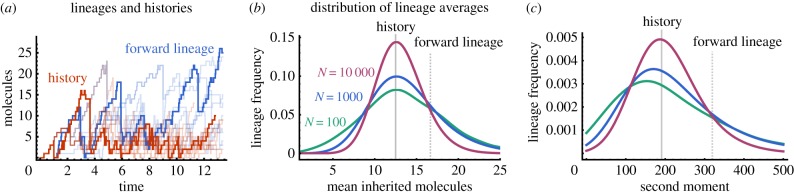
Histories represent typical lineages of finite population trees. (*a*) Representative forward lineages (blue) and histories (red lines) from a single population for the birth–death process (2.18) with rates *k*_0_ = 5 and *k*_1_ = 0.1 and division times are exponentially distributed with unit rate. Simulated time-courses show molecule synthesis by biochemical reactions (step 1 in box 1) interrupted by sudden decreases in molecule number due to binomial partitioning at cell division (step 2 in box 1). (*b*) Smoothed frequency distributions of the mean number of inherited molecules computed for each lineage of 10 population trees with *N* = 100 (teal), 1000 (blue) and 10 000 cells (magenta) at the final time and rates *k*_0_ = 50 and *k*_1_ = 1. The distribution mode is centred about the mean of histories (solid grey line) but not about the means of forward lineages (dashed grey line). (*c*) The corresponding second moment *E*[*x*^2^ | 0] is shown. The result indicates that the statistics of most lineages is close to the one of histories.

As a first application, we consider a birth–death process. Molecules are synthesized at a constant rate *k*_0_ and are degraded via a first-order reaction with rate *k*_1_,2.18
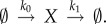
To test whether differences between forward lineages and histories could be significant in a single population, we used the First Division Algorithm (box 1) to simulate a population tree corresponding to figure 1*a*. We then select histories by choosing cells at random from the population and tracing their evolution back through the population tree. Similarly, we choose forward lineages by following the first cell in the population over many cell cycles selecting either daughter cell with equal probability. A qualitative comparison of few representative time-courses shows that forward lineages exhibit fewer cell divisions but higher molecule counts than histories (figure 3*a*), consistent with the predicted age-distributions, which contain more old cells in forward lineages than in histories (figure 2*b*).

To quantify whether histories represent typical lineages in a finite population, we simulate 40 lineage trees up to a population size of *N* = 100, 1000 and 10 000. We then calculate mean and second moments for each individual lineage in the tree and evaluate their distributions across all lineages (figure 3*b*,*c*). As the population size increases the distribution of first and second moments becomes centred about the moments of the history distribution which are smaller than the corresponding ones in forward lineages. In particular, the moments of histories correspond to the distribution modes verifying that histories are typical lineages, even for finite populations.

Next, we investigate how to analytically characterize the distributions of both processes. The generating function of the corresponding master equation obeys the evolution equation2.19
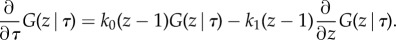
Because molecules are produced and degraded independently, their number *x* per cell can be separated into a sum of two independent contributions: (i) the amount of molecules newly produced and degraded up to age *τ*, and (ii) the amount of molecules inherited after cell division. Clearly, the first contribution is Poissonian and its mean is given by *μ*(*τ*) = (*k*_0_/*k*_1_)(1 − e^−*k*_1_*τ*^). The second contribution needs to be determined from the boundary condition as we show in the following.

The solution to equation (2.19) can be written as a product of generating functions of the aforementioned contributions2.20
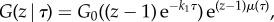
where the first factor is the generating function of newly produced and degraded molecules until age *τ*. Using this solution in equation (2.17) we find the condition2.21
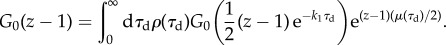
If cell divisions occur at deterministic time intervals, the integral is straightforward. In this case, the distribution of inherited molecules *p*_0_ is Poissonian with mean *μ*_p_(*τ*) = ((1 − 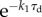)/(2 − 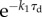))(*k*_0_/*k*_1_) 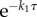 and depends on the division-time *τ*_d_. This result has been obtained earlier by Schwabe *et al.* [29], and as a particular case by Johnston *et al.* [19].

The solution turns out to be more involved in the presence of division-time variability. Certainly, equation (2.20) could be solved for particular choices of *ρ*(*τ*_d_). Since, however, the division-time variability is difficult to characterize, in general, we aim to solve this equation for arbitrary distributions. To this end, we expand2.22
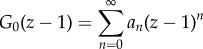
and write an equation for the series coefficients *a*_*n*_. Plugging equation (2.22) into (2.20) and equating equal powers of *z*, we find2.23
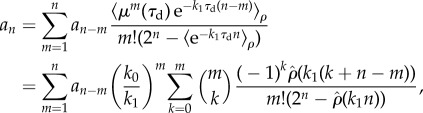
where 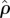 is the Laplace transform of the division-time distribution in equation (2.11*c*). From the above formula all coefficients can be computed recursively using *a*_0_ = 1. It follows using *G*_0_((*z* − 1) e^−*k*_1_^^*τ*^) in equation (2.22) and differentiating at *z* = 1 that the probability of observing *x* molecules inherited after cell division is2.24
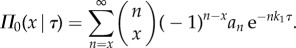
Note that the distribution depends on the cell age *τ* because the inherited molecules are degraded over the cell cycle. Because the number of molecules produced up to age *τ* is Poissonian, the total amount of molecules for a cell of age *τ* obeys2.25
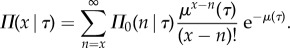
The distributions of forward lineages are obtained by substituting *φ* for *ρ* in equation (2.23) (appendix A).

**Figure 4.**
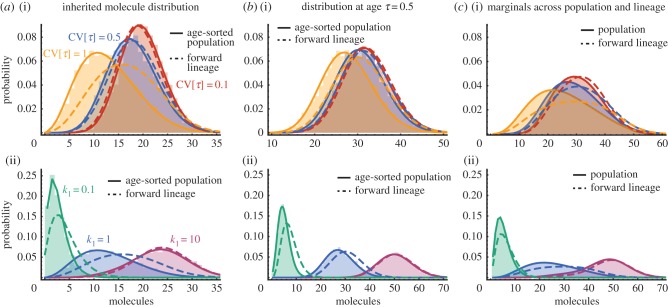
Analytical solutions for a birth–death process. (*a*)(i) Analytical distributions of inherited molecules in a population and in forward lineages are depicted for gamma-distributed division times with 10% (red), 50% (blue) and 100% (yellow line) variations about the mean (as shown in figure 2). Simulated distributions of inherited molecules in histories are shown as shaded areas. Lineages and histories agree only for small division times variability. Parameters are *k*_0_ = 50 and *k*_1_ = 1. (*a*)(ii) Analytical solutions for different degradation rates, *k*_1_ = 0.1 (teal), *k*_1_ = 1 (blue) and *k*_1_ = 10 (magenta). The distribution of inherited molecules in a population (*τ* = 0, solid lines) coincides with the simulated histories (shaded ares), which verifies the ergodic principle, but not with the lineage distribution (dashed lines). Histories and forward lineages agree well only for large degradation rates. Parameters are *k*_0_ = 50*k*_1_ and exponentially distributed division times with unit mean (CV[*τ*] = 1). (*b*) The corresponding distributions are shown for the mid-cell cycle *τ* = 0.5. (*c*) Simulated distributions estimated irrespectively of cell age across the population (shaded areas) agree well with analytical solutions (solid lines). Also shown are the theoretical distributions across forward lineages (dashed lines).

In figure 4*a*, we show the resulting distributions of inherited molecules (*τ* = 0) for different levels of cell-cycle variability (figure 4*a*,i) for the cases of an age-sorted population (solid lines) and for the lineage statistics (dashed lines). Division times are assumed to be gamma-distributed, which fits cell-cycle variability observed in many bacteria [8,10]. The molecule number distributions are obtained using either *ρ* or *φ* in equation (2.23) and truncating equation (2.24) after the first 150 terms. In agreement with the theoretical predictions, lineage and histories distributions are similar for small division time variability (red lines) but become significantly different as variability increases (yellow lines). Also shown are the corresponding distribution for varying degradation rates (figure 4*a*,ii). We observe that forward lineages and history statistics are comparable for unstable molecules (fast degradation) but not for stable ones (slow degradation). This finding is in line with the intuition that rapid degradation averages out timing fluctuations.

To support the numerical results, we evaluate the mean number of molecules at birth 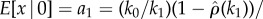 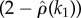. For fast degradation, the expression reduces to 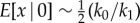 in both forward lineages and histories because it is independent of the division times. For slow degradation, however, we obtain *E*[*x* | 0] ∼ *k*_0_*E*_*ρ*_(*τ*_d_) in histories and *E*_fw_[*x* | 0] ∼ *k*_0_*E*_*φ*_(*τ*_d_) in forward lineages. Since *E*_*ρ*_(*τ*_d_) ≤ *E*_*φ*_(*τ*_d_) with equality for deterministic division times [10], we conclude that cells in histories and the population contain fewer molecules than in forward lineages. One can further show that the difference between population and forward lineages *E*[*x* | 0] − *E*_fw_[*x* | 0] = − *k*_0_ln 2 CV^2^_*φ*_[*τ*] + *O*(CV^4^_*φ*_) increases with the coefficient of variation of timing fluctuations.

In figure 4*b*, we illustrate the corresponding distributions for *τ* = 0.5 corresponding to half of the mean generation time. We observe that both cell-cycle variation (figure 4*b*,i) and degradation (figure 4*b*,ii) mitigate the discrepancies between lineage and history statistics. Intuitively, this could also be concluded from the fact that equation (2.19), and similarly the distribution of molecules equation (2.11*a*), approaches a steady state independent of the number of inherited molecules for large *τ*. For all cases, we verified the ergodic principle by stochastic simulations using the First Division Algorithm (box 1) of 40 population trees from which we chose histories at random. The corresponding distributions are shown as shaded areas in figure 4*a*,*b*, which are in excellent agreement with our analytical solutions (solid lines).

To investigate the effect of the overall distribution in the population irrespectively of cell age, we numerically integrate the analytical solution (2.25) over the age-distribution *Π*(*τ*) of the population given by equation (2.8), 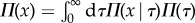; the result is shown in figure 4*c*. The corresponding age distribution in a forward lineage is given in appendix D. We find that these predictions (solid lines) are in excellent agreement with the simulated distributions across populations (shaded areas) but not with the distributions across forward lineages (dashed lines).

#### 2.2.2. Expression of a stable protein

Characteristic mRNA half-lives in *E. coli* are of the order of 5 min, while doubling times range from 20 min to several hours. Given the findings of the previous section, this suggests that generation time variability has little effect on mRNA distributions. Protein half-lives in living cells, however, occur on timescales much longer than the doubling time, meaning that many proteins are essentially stable and diluted mainly through cell divisions.

**Figure 5.**
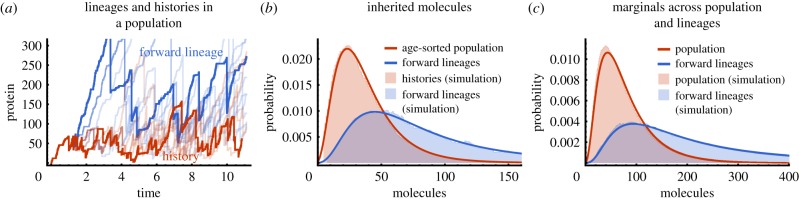
Lineage and history characterization of the expression of a stable protein. (*a*) Representative time courses of forward lineages (blue) and histories (red) of a stable protein collected from a single population tree. (*b*) Simulated distributions of inherited molecules in forward lineages (blue) and histories (red) are shown as shaded areas. The analytical solution for a population (solid red line) agrees with the simulated histories but not with the lineage verifying the ergodic principle. For comparison, we also show the analytical solution of a lineage (solid blue line). (*c*) Distributions obtained irrespectively of cell age from forward lineages (shaded blue) and across the population (shaded blue), both of which are in excellent agreement with the simulations. In all panels, the distribution of division time is lognormal with unit mean and s.d. 3 resulting in a growth rate of approximately 1.25. Parameters for the simulation of the full gene expression model (2.26) are *k*_0_ = 20, *k*_1_ = 20 and *k*_2_ = 100.

To investigate protein variability in growing cell populations, we model the expression of such a protein including mRNA transcription via the reactions2.26
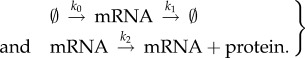
Using the First Division Algorithm (box 1), we simulate a population of dividing cells and collect representative forward lineages and histories (figure 5*a*). Given that empirical distributions of rescaled log-division times in *E. coli* are invariant and bell-shaped across conditions [46], we assume lognormal distributed division times. We observe that protein time-courses corresponding to forward lineages have higher protein levels and are noisier than histories.

Deriving analytical expressions for the distribution of proteins is more involved because the amount of produced protein generally depends on the amount of inherited mRNA. We use the simplifying assumption that the mRNA half-life is much shorter than the cell-cycle time, for which the reactions (2.26) reduce to a single reaction [47,48]2.27
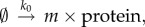
with proteins being produced in stochastic bursts of size *m* with distribution *ν*_*m*_. For the reactions (2.26), the burst size distribution *ν*_*m*_ is geometric with mean *b* = *k*_2_/*k*_1_ corresponding to the mean number of proteins produced per mRNA lifetime [48].

The rate of change for the generating function of this process satisfies2.28
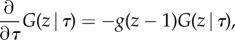
where 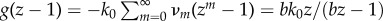. It can be verified using equation (2.28) and the boundary condition (2.17) that the exact generating function solution is2.29
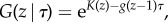
where 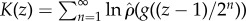 and 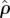 is the Laplace transform of *ρ*. Marginalizing the above equation over the age distribution, we obtain the generating function for the protein number distribution in the population2.30
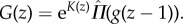
An explicit expression for the Laplace transform of the age-distribution 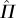 is given in appendix D, equation (D 3).

The distributions corresponding to the generating function, equation (2.29), are obtained numerically via the inverse transform2.31
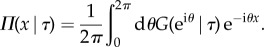
Note that because the moment-generating function does not exist for the lognormal distribution, we computed the Laplace transform of the division-time distribution directly. In practise, it was sufficient to truncate the sum in *K*(*z*) after the first 10 terms. In figure 5*b*, we show the resulting distribution of inherited protein in a population (*τ* = 0, red line). We observe excellent agreement between the theoretical solution and the history statistics obtained from stochastic simulations of 40 population trees (shaded red area), which verifies the ergodic principle. Instead, the distribution obtained across forward cell lineages shows a broader distribution with significant distribution tails (blue line) due to larger division time variability, in agreement with simulations (shaded blue area). Similar to the previous example, cells in histories and the population inherit fewer molecules than in forward lineages. We also obtain good agreement with the snapshot distributions measured across the cell population shown in figure 5*c* (red line: theory, red shaded area: simulation) by instead using equation (2.30) in the transformation (2.31).

### 2.3. Breakdown of the ergodic principle under selection

**Figure 6.**
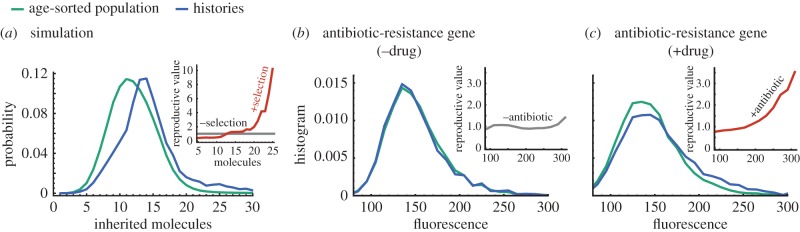
The ergodic principle breaks down in the presence of selection. (*a*) Simulated distributions of inherited molecules for a biomolecule that increases division rate. It is seen that histories and age-sorted distributions are different and thus the ergodic principle is violated. The ratio of these probabilities yields the reproductive value, *ν*(*x* | 0), which increases with the number of molecules in the presence of selection (inset). In the simulation molecules are produced with rate *k*_0_ and divisions occur after a period *τ*_d_ = 50*K*^10^/(*K*^10^ + *x*^10^_0_), where *k*_0_ = 100, *K* = 10 and *x*_0_ is the molecule count of that cell at birth. (*b*) Statistics of tree data of an antibiotic-resistance gene in the absence of antibiotic taken from Nozoe *et al.* [42]. The history distribution agrees very well with the population distribution. The ergodic principle is obeyed and the reproductive value of cells is independent of the gene expression (inset). (*c*) By contrast, in the presence of the drug, the distributions of histories and the age-sorted population disagree. The increase of the reproductive value with fluorescence indicates selection on high protein levels (inset).

So far, we considered traits that do not affect the cell division rate. To test the validity of the principle in the opposite case, we simulate the production of a biomolecule whose levels increase division rate. We find that the distributions in histories do not coincide with the age-sorted population (figure 6*a*). The history distribution exhibits significantly larger tails resulting in a higher mean number of molecules (21.6 in histories versus 12.4 in population). This breakdown suggests that one could in principle discriminate the factors that affect cell fitness.

To investigate this effect, we consider *Fisher's* reproductive value [49], denoted by *ν*, which counts the relative number of future offspring for a cell in a given phenotypic state. Interestingly, it can be defined as the ratio of the distribution of histories and the age-sorted population statistics [50]. For a given trait *x* observed in cells of age *τ*, we here use their conditional distributions that provide the following factorization2.32
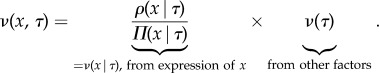
The first factor is the reproductive value due expression of *x*, involving the distributions across the age-sorted population *Π*(*x* | *τ*) and across histories *ρ*(*x* | *τ*). The second factor, *ν*(*τ*) = *Π*_*h*_(*τ*)/*Π*(*τ*), denotes the contribution from other factors affecting the division rate and is given by the ratio of the age distribution of histories *Π*_*h*_(*τ*) and the age distribution of the population *Π*(*τ*), which are generally different [10,40]. According to the ergodic principle the first contribution equals 1, when *x* does not affect the division rate, and thus the reproductive value is solely determined by ‘other’ factors that are not explicitly known and modelled via a stochastic interdivision time. When *x* increases division rate, higher levels of *x* can be selected upon and the reproductive value *ν*(*x* | *τ*) increases with *x* (figure 6*a*, inset). Thus, when the molecule levels are selected upon, ancestral cells contain more molecules on average than cells of the present population (figure 6*a*).

We investigate this dependence using recently acquired tree data of the antibiotic-resistance gene smR conveying resistance to the antibiotic streptomycin [42]. The protein is fused to a fluorescent reporter allowing direct observation. We obtain age-sorted distributions using measured total fluorescence after cell division (*τ* = 0), while lineage-weighted distributions are computed using equations (2.13) and (2.14). In the absence of antibiotic treatment, the distributions of new-born cells in the age-sorted, histories and forward lineages are essentially the same (figure 6*b*). In agreement with the ergodic principle, the reproductive value due to the expression of the protein is constant (figure 6*b*, inset). In the presence of sub-inhibitory antibiotic doses, the distribution in histories differ from the one acquired across the population (figure 6*c*) and, moreover, the reproductive value increases with fluorescence (inset), which indicates selection on higher expression levels. These results are in agreement with the known function of the gene and highlight that testing the proposed ergodic principle can be used to probe selection in cell populations.

## 3. Discussion

We presented a modelling approach for stochastic biochemical reactions in dividing cell populations. The theory enabled us to identify an ergodic principle between single cells and the population, which can be stated as the average of the histories of single cells in a population obtained over many cell divisions equals the average over an age-sorted cell population at any single time point. Cell histories capture the statistics of typical lineages in a population, which are obtained by choosing an arbitrary individual of a clonal population and tracking back its evolution to the ancestor from which the population originated.

This principle provides an interpretation of population snapshot data because it identifies the sample paths that are associated with age-sorted snapshot data with typical cell histories. Histories thus allow us to characterize the progression of single cells in the population through the cell cycle. We emphasized that the statistical difference between forward lineages and histories arises from variable division times, cells dividing faster than the population growth rate being over-represented in histories as compared to forward lineages. These deviations are expected to be significant since generation times of single cells are highly variable in microbial cell populations.

It is important to point out that the principle requires the cell age to be known. The exception to this rule is the case of exponential division times for which the time-average of histories equals the one across snapshots. In the general case, gating techniques as used in flow cytometry can be used to narrow the range of physiological differences and thus correspond to age-sorted distributions, which according to the principle agree with the histories of single cells. Mother machines, on the other hand, provide statistics of individual forward lineages assuming that cells divide symmetrically. Thus our findings suggest potential differences when studying the dynamics of gene expression in different experimental devices, even when cell-cycle positions are explicitly known.

Different notions of ergodicity have been discussed in the literature. Rocco *et al.* [17], for instance, challenged the ergodic hypothesis between an ensemble of individual lineages and their time-averages. They found that the hypothesis only holds true for fast degrading molecules. We found a similar dependence for the ergodicity between snapshots of a growing population and individual histories, which is justified when studying the dynamics of short-lived molecules such as mRNAs. The majority of proteins in *E. coli* and yeast, however, have half-lives much longer than typical population doubling times [51,52], for which the present ergodic principle accounts for. We demonstrated this dependence using analytical solutions to lineage, history and population distributions of simple stochastic models of gene expression.

Although we developed the approach for biochemical reactions, the general principle applies to heritable non-genetic traits, such as plasmid numbers or chromatin states. Specifically, it applies to any trait of interest that does not affect the cell division rate. A counterexample of such a trait is cell size to which the history analysis has recently been applied [43]. Here, we found the that the likelihood to find an ancestral cell with a positively selected trait value is higher than for cells of the present population. Thus testing the ergodic principle could be used to experimentally probe this effect on cell fitness for any trait of interest.

## Data accessibility

This article has no additional data.

## Appendix A. Biochemical dynamics in forward lineages

The lineage description tracks a single cell over many cell division cycles but retains information about only one daughter at each division. We consider the lineage-probability *p*_*D*_(*x*|*τ*) of observing *x* molecules in a cell of age *τ* after *D*th division. In between divisions the distribution satisfies the chemical master equation

A 1*a*

where the transition matrix is defined in equation (2.3*b*) of the main text. An important difference to the usual master equation is that it needs to be solved subject to both an initial condition *p*_0_(*x* | 0) and a boundary condition describing cell divisions. The latter is given by

A 1*b*

where *φ*(*τ*_d_) is the distribution of division times, *B*(*x* | *x*′) is the division kernel as defined in equation (2.4) of the main text, and the summation is over all possible molecular states *x*′. The stationary lineage-probability is obtained in the long-term limit *p*(*x* | *τ*) = lim_*D* → ∞_*p*_*D*_(*x* | *τ*) after a large number of cell divisions, or equivalently, by letting *p*_*D*_(*x* | *τ*) = *p*_*D*+1_(*x* | *τ*) in equation (A 1). The boundary condition, equation (A 1*b*), couples the distribution of dividing to the one of newborns.

## Apendix B. Master equation description of snapshot probabilities and snapshot densities

We here derive a stochastic description for the outcome of snapshots in a dividing cell population. The state of the system may be described by observations of molecule content and cellular age {*τ*_*i*_, *x*_*i*_}_*i*=*N*_, where *N* is the present number of cells. Note that *τ*_*i*_ and *x*_*i*_ but also *N* are random variables for a single snapshot [9]. When cells are indistinguishable, a snapshot is equivalently characterized by the empirical distribution function

B 1

Generally, more than a single snapshot is available, for instance by collecting snapshots from different microcolonies. Aggregating snapshots averages out the dependence on the individual stochastic realizations of {*τ*_*i*_, *x*_*i*_}_*i*=*N*_, and yields the snapshot density

B 2

quantifying the frequency with which cells with molecule count *x* and age *τ* are observed in the population. In the following, we first derive a master equation description for the functional probabilities *P*[*s*, *t*] of observing a particular realization of the snapshot *s*(*x*, *τ*) at time *t*. Second, we perform the average over all possible snapshots to obtain the density *n*(*x*, *τ*). An alternative approach to the one presented here would extend the kinetic framework given in [53] to incorporate biochemical reactions.

We consider two types of transitions: (i) cell division in which molecules are partitioned between the two daughter cells and (ii) biochemical reactions. The rate of cell division for a single cell of age *τ*′ is *γ*(*τ*′) and hence the propensity of cell divisions in the population is

B 3

At division, the mother cell is replaced with two new cells of zero age. The snapshot, therefore, changes instantaneously by

B 4

where *τ*′ and *x*′ are the respective age and molecule count of the mother cell, and *x*_1_ and *x*_2_ the molecule counts of the daughter cells.

When a biochemical reaction with stoichiometry *ν*_*r*_ occurs, we replace the mother cell with molecule count *x*′ by a cell of the same age but *x*′ + *ν*_*r*_ molecules. The snapshot therefore changes as follows:

B 5

For brevity of notation, we define the step-operator, which acts as  on any functional *f* of the snapshot function *s*(*x*, *τ*). Combining the changes from divisions and *R* possible reactions, we obtain the change in the snapshot probability *P*[*s*, *t*], describing the probability of all possible snapshots, which obeys

where we assume that *B*(*x*_1_, *x*_2_ | *x*′) is the probability of partitioning *x*′ molecules into the proportions *x*_1_ and *x*_2_ of the daughter cells.

The snapshot density *n*(*x*, *τ*, *t*) from repeated experiments is obtained as follows:

Thus multiplying equation (B 6) by *s*(*x*, *τ*), integrating over all possible snapshots, and using the definition of the total derivative, we obtain the master equation for the snapshot density

where  and   are the marginal distributions of the partition probability. If the molecule numbers are conserved during cell division, we must have *B*_2_(*x* | *x*′) = *B*_1_(*x*′ − *x* | *x*′).

Integrating the above equation over a small interval  containing the newborn cells and using *n*(*x*, *ε*) → *n*(*x*, 0), *n*(*x*, − *ε*) → 0 and definition (2.4) of the main text, we find that the second and third term on the right-hand side of the above equation give the boundary condition (2.3c) of the main text. The remaining terms describe the rate of change in between divisions and are equal to equation (2.3a) with (2.3b) of the main text.

## Appendix C. Extension to heritable interdivision times

We here discuss an extension of the ergodic principle. We consider a general trait *x* and division times that are statistically dependent between mother daughter cells. Such correlations could arise, for instance, due to heritable factors such as cell size. To proceed we characterize the state of each cell by a cell age *τ* and a interdivision time *τ*_d_. The latter is a random quantity that yields the age at which a cell will eventually divide. The distribution of cell ages at division must then satisfy

C 1

for an appropriate choice of the division rate *γ*(*s*, *τ*_d_).

### C.1. Forward lineages

At stationarity, the division time distribution along a forward lineage can then be described by

C 2

Similar to the case of independent division times, the lineage dynamics of the trait *x* is a function of cell age

C 3

but does not depend on the future division time. The boundary condition describing cell divisions is

C 4

Note that for generality we have included the case where the division kernel *B*(*x* | *x*′, *τ*_d_) also depends on heritable factors effectively through its dependence on the age at division *τ*_d_.

### C.2. Distribution of age and division times

For the cell population, we describe the density of cells with age *τ*, future division time *τ*_d_ and the trait *x*, which follows the evolution equation

C 5*a*

To account for cell divisions, we supplement the above equation by a boundary condition, which again replaces each mother cell by two daughter cells of zero age but with the trait *x*′ replaced by *x* after cell division. The latter can be expressed as

C 5*b*

Summing equation (C 5a) over all possible trait values *x* and solving for *Π*(*τ*, *τ*_d_), we find that the age-division-time distribution is

C 6

where *θ* denotes the Heaviside function. The division times of newborn cells in the population follow *Π*(*τ*_d_ | 0), which is obtained from

C 7

The constant *Π*(0) can be determined by integrating equations (C 5) over all possible values of *τ*, *τ*_d_ and *x* and combining the results to obtain

C 8

Specifically, marginalizing equation (C 6) over the division times *τ*_d_, the age distribution becomes

C 9

Further, summing equation (C 5*b*) over all possible values of *x* and combing equations (C 1), (C 6) and (C 7) with the boundary condition (C 5*b*) gives an integral equation for the division time of newborn cells in the population

C 10

Interestingly, when division time is inherited this distribution is different from the one in a forward lineage. The above equation has first been derived by Powell [8] to investigate the effect of mother–daughter correlations, albeit using a different approach. The population growth rate is implicitly defined through the normalizing condition

C 11

In particular, we note that the distribution below the integral is the ancestral division time distribution

C 12

This distribution counts all divisions that occurred in a stationary population up to a certain point in time. The distribution of ‘future’ division times is *Π*(*τ*_d_)

C 13

which satisfies the relation

C 14

### C.3. Distribution of a trait along histories

Finally, we turn our attention to the distribution of molecule numbers for cells of a given age. To this end, it is important to note that the molecule numbers are non-anticipating in that they are statistically independent of division time *τ*_d_ but they only depend on cell age

C 15

It is worth pointing out that this statement assumes the division times to be independent of the trait of interest. In effect, the distribution of molecules at a certain cell age evolves according to

C 16*a*

The boundary condition can be derived by letting *Π*(*τ*, *x*, *τ*_d_) = *Π*(*x* | *τ*)*Π*(*τ*, *τ*_d_) in equation (C 5*b*), which gives

C 16*b*

We observe that this is the same equation as obtained in a forward lineage but replacing the lineage distribution of division times with the ancestral distribution *ρ*(*τ*_d_), equation (C 12). Because histories contain only ancestral cells, the above equation also determines the distribution in cell histories. In summary, we have shown that the distribution of a cellular trait along a history equals the distribution in a dividing cell population with inherited division times.

## Appendix D. Properties of the age distributions

Consider an age distribution of the form

D 1

which for *λ* = 0 coincides with the age distribution in forward lineages and for *λ*≠0 with the age distribution in a population. Its Laplace transform  can be expressed as follows:

D 2

where  denotes the Laplace transform of the division-time distribution *φ*. For the population, it follows from  and  that *Π*(0) = 2*λ*. Hence, we obtain the Laplace transform

D 3

For forward lineages, we use *λ* = 0 in equation (D 1) and the fact that

D 4

equals the average division time. It then follows that *Π*(0) = 1/〈*τ*〉 and hence

D 5

or, equivalently,

D 6

## References

[1] McAdams HH, Arkin A 1997 Stochastic mechanisms in gene expression. Proc. Natl Acad. Sci. USA 94, 814–819. (10.1073/pnas.94.3.814) 9023339 PMC19596

[2] Oyarzún DA, Lugagne J-B, Stan G-BV 2014 Noise propagation in synthetic gene circuits for metabolic control. ACS Synth. Biol. 4, 116–125. (10.1021/sb400126a) 24735052

[3] Martins BM, Locke JC 2015 Microbial individuality: how single-cell heterogeneity enables population level strategies. Curr. Opin. Microbiol. 24, 104–112. (10.1016/j.mib.2015.01.003) 25662921

[4] Zechner C, Seelig G, Rullan M, Khammash M 2016 Molecular circuits for dynamic noise filtering. Proc. Natl Acad. Sci. USA 113, 4729–4734. (10.1073/pnas.1517109113) 27078094 PMC4855548

[5] Gillespie DT 1976 A general method for numerically simulating the stochastic time evolution of coupled chemical reactions. J. Comput. Phys. 22, 403–434. (10.1016/0021-9991(76)90041-3)

[6] Elowitz MB, Levine AJ, Siggia ED, Swain PS 2002 Stochastic gene expression in a single cell. Science 297, 1183–1186. (10.1126/science.1070919) 12183631

[7] Thomas P, Popović N, Grima R 2014 Phenotypic switching in gene regulatory networks. Proc. Natl Acad. Sci. USA 111, 6994–6999. (10.1073/pnas.1400049111) 24782538 PMC4024914

[8] Powell E 1956 Growth rate and generation time of bacteria, with special reference to continuous culture. Microbiology 15, 492–511. (10.1099/00221287-15-3-492) 13385433

[9] Stukalin EB, Aifuwa I, Kim JS, Wirtz D, Sun SX 2013 Age-dependent stochastic models for understanding population fluctuations in continuously cultured cells. J. R. Soc. Interface 10, 20130325 (10.1098/rsif.2013.0325) 23760298 PMC3971721

[10] Hashimoto M, Nozoe T, Nakaoka H, Okura R, Akiyoshi S, Kaneko K, Kussell E, Wakamoto Y 2016 Noise-driven growth rate gain in clonal cellular populations. Proc. Natl Acad. Sci. USA 113, 3251–3256. (10.1073/pnas.1519412113) 26951676 PMC4812751

[11] Volfson D, Marciniak J, Blake WJ, Ostroff N, Tsimring LS, Hasty J 2006 Origins of extrinsic variability in eukaryotic gene expression. Nature 439, 861–864. (10.1038/nature04281) 16372021

[12] Hilfinger A, Paulsson J 2011 Separating intrinsic from extrinsic fluctuations in dynamic biological systems. Proc. Natl Acad. Sci. USA 108, 12 167–12 172. (10.1073/pnas.1018832108) 21730172 PMC3141918

[13] Paulsson J 2004 Summing up the noise in gene networks. Nature 427, 415–418. (10.1038/nature02257) 14749823

[14] Swain PS, Elowitz MB, Siggia ED 2002 Intrinsic and extrinsic contributions to stochasticity in gene expression. Proc. Natl Acad. Sci. USA 99, 12 795–12 800. (10.1073/pnas.162041399) PMC130539 12237400

[15] Lu T, Volfson D, Tsimring L, Hasty J 2004 Cellular growth and division in the Gillespie algorithm. IET Syst. Biol. 1, 121–128. (10.1049/sb:20045016) 17052122

[16] Huh D, Paulsson J 2011 Random partitioning of molecules at cell division. Proc. Natl Acad. Sci. USA 108, 15 004–15 009. (10.1073/pnas.1013171108) PMC3169110 21873252

[17] Rocco A, Kierzek AM, McFadden J 2013 Slow protein fluctuations explain the emergence of growth phenotypes and persistence in clonal bacterial populations. PLoS ONE 8, e54272 (10.1371/journal.pone.0054272) 23382887 PMC3558523

[18] Bierbaum V, Klumpp S 2015 Impact of the cell division cycle on gene circuits. Phys. Biol. 12, 066003 (10.1088/1478-3975/12/6/066003) 26403517

[19] Johnston IG, Jones NS 2015 Closed-form stochastic solutions for non-equilibrium dynamics and inheritance of cellular components over many cell divisions. Proc. R. Soc. A 471, 20150050 (10.1098/rspa.2015.0050) 26339194 PMC4550007

[20] Wang P, Robert L, Pelletier J, Dang WL, Taddei F, Wright A, Jun S 2010 Robust growth of *Escherichia coli*. Curr. Biol. 20, 1099–1103. (10.1016/j.cub.2010.04.045) 20537537 PMC2902570

[21] Spivey EC, Jones SK Jr, Rybarski JR, Saifuddin FA, Finkelstein IJ 2017 An aging-independent replicative lifespan in a symmetrically dividing eukaryote. eLife 6, e20340 (10.7554/eLife.20340) 28139976 PMC5332158

[22] Kiviet DJ, Nghe P, Walker N, Boulineau S, Sunderlikova V, Tans SJ 2014 Stochasticity of metabolism and growth at the single-cell level. Nature 514, 376–379. (10.1038/nature13582) 25186725

[23] Antunes D, Singh A 2015 Quantifying gene expression variability arising from randomness in cell division times. J. Math. Biol. 71, 437–463. (10.1007/s00285-014-0811-x) 25182129

[24] Soltani M, Vargas-Garcia CA, Antunes D, Singh A 2016 Intercellular variability in protein levels from stochastic expression and noisy cell cycle processes. PLoS Comput. Biol. 12, e1004972 (10.1371/journal.pcbi.1004972) 27536771 PMC4990281

[25] Cerulus B, New AM, Pougach K, Verstrepen KJ 2016 Noise and epigenetic inheritance of single-cell division times influence population fitness. Curr. Biol. 26, 1138–1147. (10.1016/j.cub.2016.03.010) 27068419 PMC5428746

[26] Shen F, Hodgson L, Rabinovich A, Pertz O, Hahn K, Price JH 2006 Functional proteometrics for cell migration. Cytometry A 69, 563–572. (10.1002/cyto.a.20283) 16752422

[27] Newman JR, Ghaemmaghami S, Ihmels J, Breslow DK, Noble M, DeRisi JL, Weissman JS 2006 Single-cell proteomic analysis of *S. cerevisiae* reveals the architecture of biological noise. Nature 441, 840–846. (10.1038/nature04785) 16699522

[28] So L-H, Ghosh A, Zong C, Sepúlveda LA, Segev R, Golding I 2011 General properties of transcriptional time series in *Escherichia coli*. Nat. Genet. 43, 554–560. (10.1038/ng.821) 21532574 PMC3102781

[29] Schwabe A, Bruggeman FJ 2014 Contributions of cell growth and biochemical reactions to nongenetic variability of cells. Biophys. J. 107, 301–313. (10.1016/j.bpj.2014.05.004) 25028872 PMC4104058

[30] Kafri R, Levy J, Ginzberg MB, Oh S, Lahav G, Kirschner MW 2013 Dynamics extracted from fixed cells reveal feedback linking cell growth to cell cycle. Nature 494, 480–483. (10.1038/nature11897) 23446419 PMC3730528

[31] Skinner SO, Xu H, Nagarkar-Jaiswal S, Freire PR, Zwaka TP, Golding I 2016 Single-cell analysis of transcription kinetics across the cell cycle. eLife 5, e12175 (10.7554/eLife.12175) 26824388 PMC4801054

[32] Gut G, Tadmor MD, Pe'er D, Pelkmans L, Liberali P 2015 Trajectories of cell-cycle progression from fixed cell populations. Nat. Methods 12, 951–954. (10.1038/nmeth.3545) 26301842 PMC6004611

[33] Wheeler RJ 2015 Analyzing the dynamics of cell cycle processes from fixed samples through ergodic principles. Mol. Biol. Cell 26, 3898–3903. (10.1091/mbc.E15-03-0151) 26543196 PMC4710220

[34] Huang S 2009 Non-genetic heterogeneity of cells in development: more than just noise. Development 136, 3853–3862. (10.1242/dev.035139) 19906852 PMC2778736

[35] Taniguchi Y, Choi PJ, Li G-W, Chen H, Babu M, Hearn J, Emili A, Xie XS 2010 Quantifying *E. coli* proteome and transcriptome with single-molecule sensitivity in single cells. Science 329, 533–538. (10.1126/science.1188308) 20671182 PMC2922915

[36] Brenner N, Braun E, Yoney A, Susman L, Rotella J, Salman H 2015 Single-cell protein dynamics reproduce universal fluctuations in cell populations. Eur. Phys. J. E 38, 102 (10.1140/epje/i2015-15102-8) 26410847

[37] Lin J, Amir A 2017 The effects of stochasticity at the single-cell level and cell size control on the population growth. (http://arxiv.org/abs/1611.07989).

[38] Rochman N, Popescu D, Sun SX 2017 Erg(r)odicity: hidden bias and the growthrate gain. (http://arxiv.org/abs/1706.09478).

[39] Georgii H-O, Baake E 2003 Supercritical multitype branching processes: the ancestral types of typical individuals. Adv. Appl. Prob. 35, 1090–1110. (10.1017/S0001867800012751)

[40] Wakamoto Y, Grosberg AY, Kussell E 2012 Optimal lineage principle for age-structured populations. Evolution 66, 115–134. (10.1111/j.1558-5646.2011.01418.x) 22220869

[41] Sughiyama Y, Kobayashi TJ 2017 Steady-state thermodynamics for population growth in fluctuating environments. Phys. Rev. E 95, 012131 (10.1103/PhysRevE.95.012131) 28208406

[42] Nozoe T, Kussell E, Wakamoto Y 2017 Inferring fitness landscapes and selection on phenotypic states from single-cell genealogical data. PLoS Genet. 13, e1006653 (10.1371/journal.pgen.1006653) 28267748 PMC5360348

[43] Thomas P 2017 Single-cell histories in growing populations: relating physiological variability to population growth. bioRxiv . (10.1101/100495).

[44] Rosenfeld N, Young JW, Alon U, Swain PS, Elowitz MB 2005 Gene regulation at the single-cell level. Science 307, 1962–1965. (10.1126/science.1106914) 15790856

[45] Painter P, Marr A 1968 Mathematics of microbial populations. Annu. Rev. Microbiol. 22, 519–548. (10.1146/annurev.mi.22.100168.002511) 4879521

[46] Kennard AS, Osella M, Javer A, Grilli J, Nghe P, Tans SJ, Cicuta P, Lagomarsino MC 2016 Individuality and universality in the growth-division laws of single *E. coli* cells. Phys. Rev. E 93, 012408 (10.1103/PhysRevE.93.012408) 26871102

[47] Friedman N, Cai L, Xie XS 2006 Linking stochastic dynamics to population distribution: an analytical framework of gene expression. Phys. Rev. Lett. 97, 168302 (10.1103/PhysRevLett.97.168302) 17155441

[48] Shahrezaei V, Swain PS 2008 Analytical distributions for stochastic gene expression. Proc. Natl Acad. Sci. USA 105, 17 256–17 261. (10.1073/pnas.0803850105) PMC2582303 18988743

[49] Fisher RA 1930 The genetical theory of natural selection: a complete variorum edition. Oxford, UK: Oxford University Press.

[50] Charlesworth B 1994 Evolution in age-structured populations, vol. 2 Cambridge, UK: Cambridge University Press.

[51] Maurizi M 1992 Proteases and protein degradation in *Escherichia coli*. Experientia 48, 178–201. (10.1007/BF01923511) 1740190

[52] Christiano R, Nagaraj N, Fröhlich F, Walther TC 2014 Global proteome turnover analyses of the yeasts *S. cerevisiae* and *S. pombe*. Cell Rep. 9, 1959–1965. (10.1016/j.celrep.2014.10.065) 25466257 PMC4526151

[53] Greenman CD, Chou T 2016 Kinetic theory of age-structured stochastic birth-death processes. Phys. Rev. E 93, 012112 (10.1103/PhysRevE.93.012112) 26871029

